# Supplementary material for: Structure of dimeric lipoprotein lipase reveals a pore adjacent to the active site
Source: Nat Commun. 2023 May 4;14:2569. doi: 10.1038/s41467-023-38243-9 (PMC10160067; doi:10.1038/s41467-023-38243-9)
Supplement: Supplementary file 1 — Supplementary Information [file 41467_2023_38243_MOESM1_ESM.pdf]

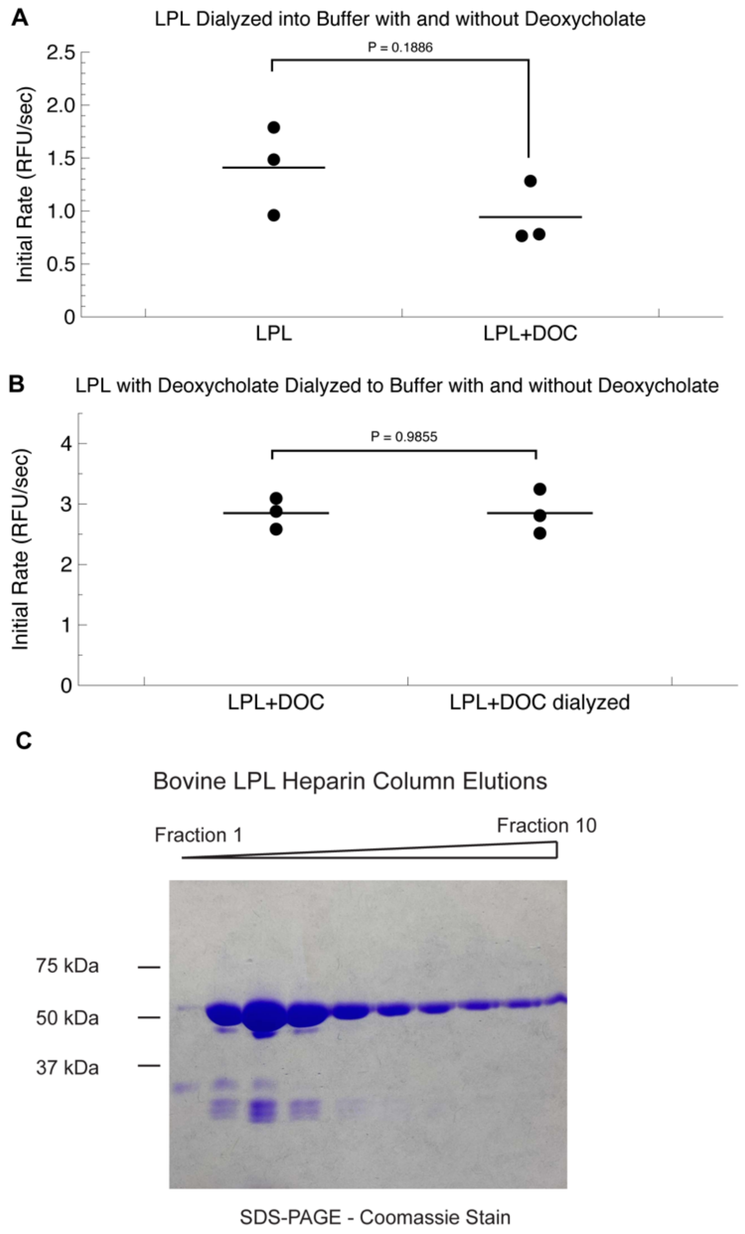

Supplementary Figure 1 – Bovine LPL activity and purification

A) LPL activity is slightly reduced following dialysis into buffer containing deoxycholate (DOC) compared to a buffer without deoxycholate. B) LPL incubated with deoxycholate and then dialyzed into a buffer with or without deoxycholate – to remove excess deoxycholate from the protein sample – have virtually identical activity. Therefore the slight change in LPL activity seen with deoxycholate is not a result of excess deoxycholate in solution. Assays were performed with 10 nM LPL and 200 μg/mL triglycerides with VLDL as technical triplicates. Each point on the graph represents a biological replicate and significance between conditions was calculated using a two-sided student's t-test. C) Coomassie stained SDS-PAGE of bovine LPL purification showing the purity of the preparation. This gel is representative of 5 separate protein purifications. Source data are provided as a Source Data file.

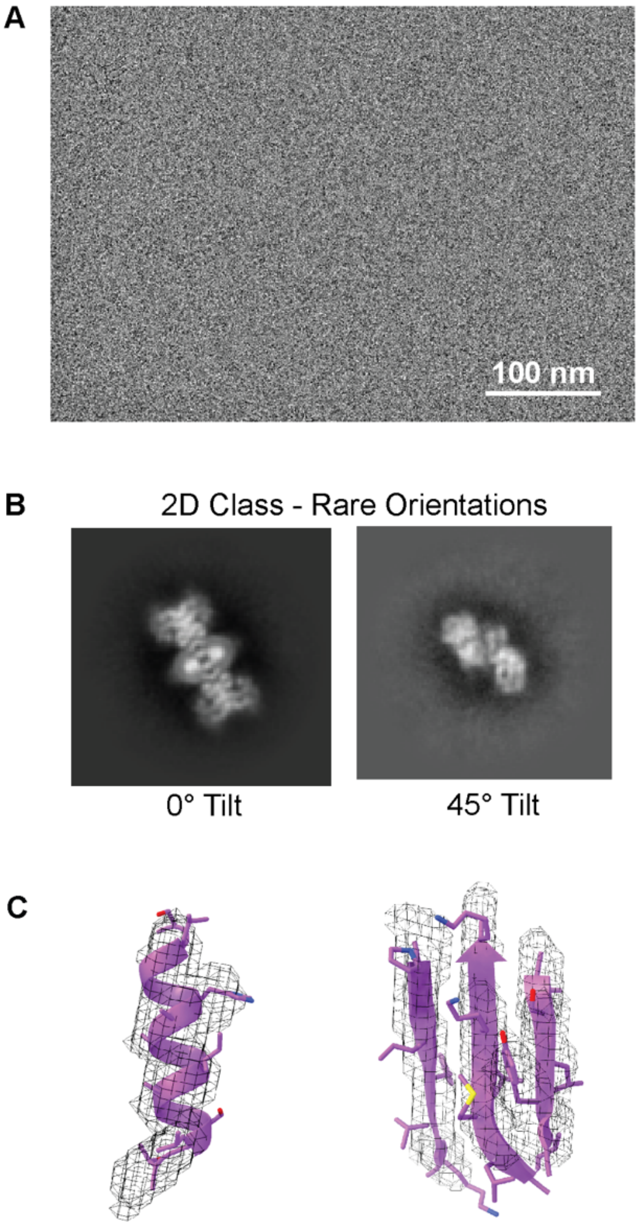

Supplementary Figure 2 – CryoEM data, 2D classes, and density fit for the LPL dimer

A) Representative micrograph for single particle LPL homodimer data. B) Rare 2D classes at each tilt were found using Topaz picking - and combined with the preferred orientation particles to solve the structure. C) The density map had clear separation between the beta-sheets and the turns of the alpha-helices are visible, as can be seen from fitting a portion of the molecular model (purple) into the density displayed as a mesh.

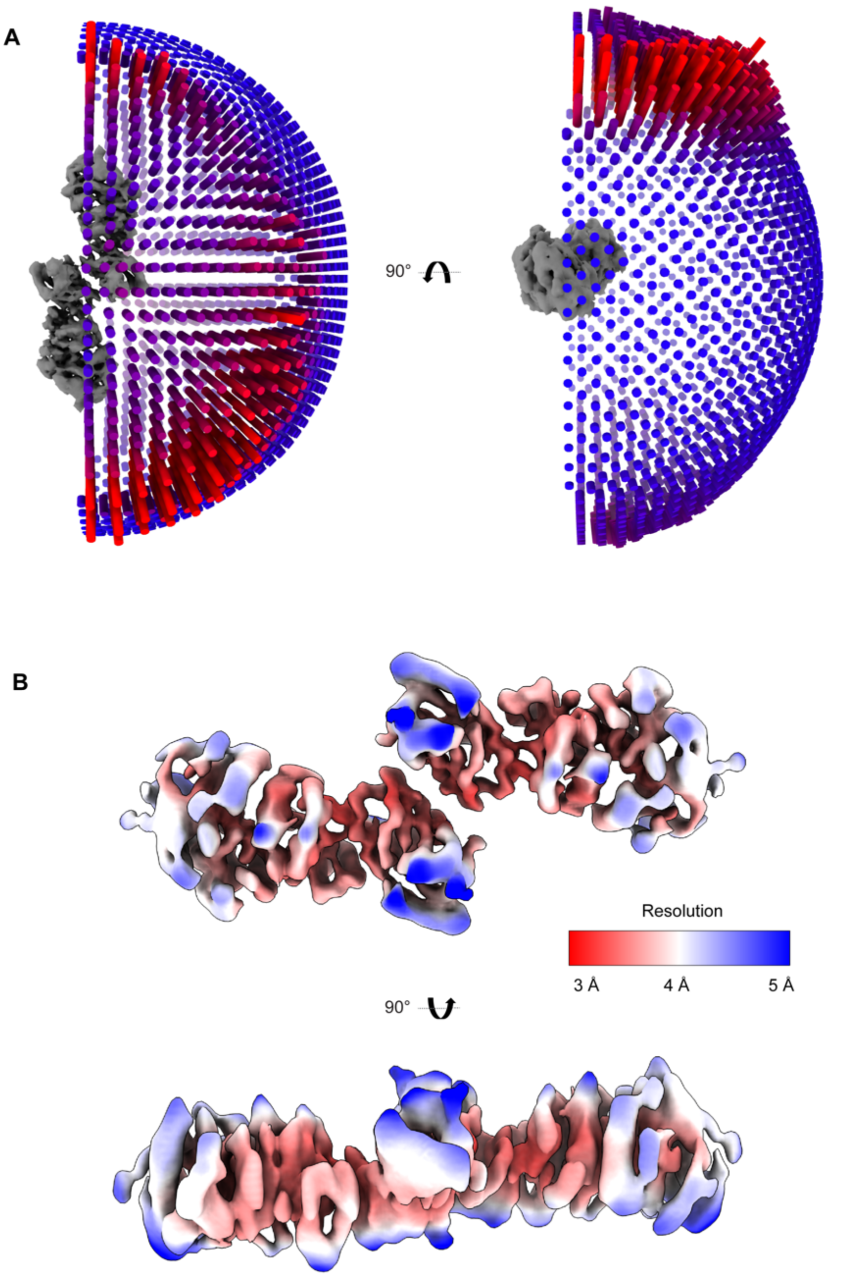

Supplementary Figure 3 – LPL particle distribution and local resolution

A) Distribution of particles compared to the resulting 3D structure of LPL. The height and color (taller and redder indicate more particles) at each spot indicates the number of particles in the final model that came from a particular angular view. This is displayed as a half sphere due to the C2 symmetry of the dimer. B) A local resolution map of the LPL dimer structure, where colors correspond to the resolution heat map in the legend. Blue indicates lower resolution, red higher.

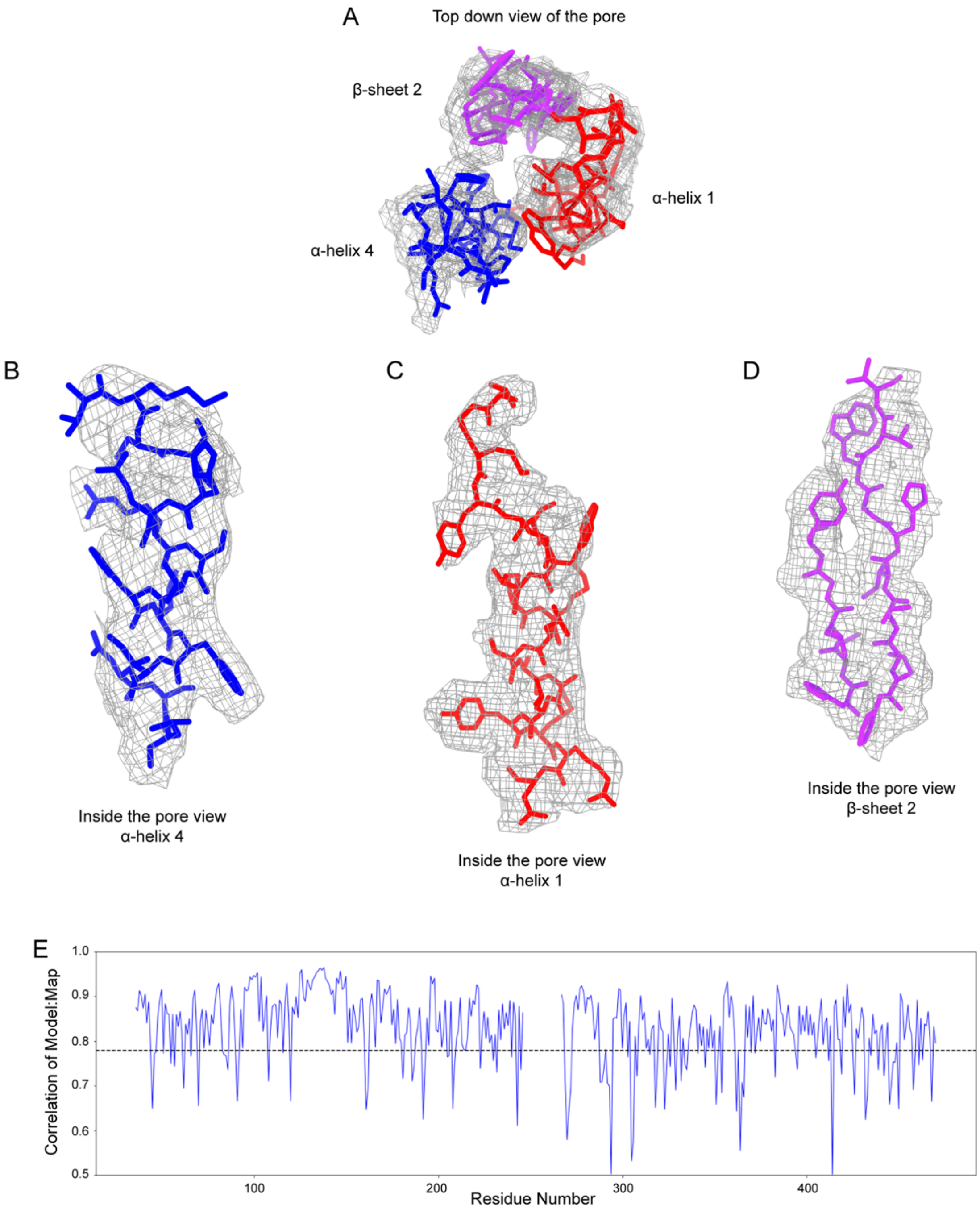

Supplementary Figure 4 – Fit of the LPL residues from the hydrophobic pore to the map

A) A view of the pore from the top with the three component secondary structures of the pore colored. B) The fit of α-helix 4 residues (blue) into the cryoEM density (mesh). C) The fit of α-helix 1 residues (red) into the cryoEM density (mesh). D) The fit of the first two strands of β-sheet 2 residues (purple) into the cryoEM density (mesh). E) The correlation between the cryoEM density and each residue of the model built into the cryoEM map. The average cross correlation of 0.77 is represented by a dotted line. Source data are provided as a Source Data file.

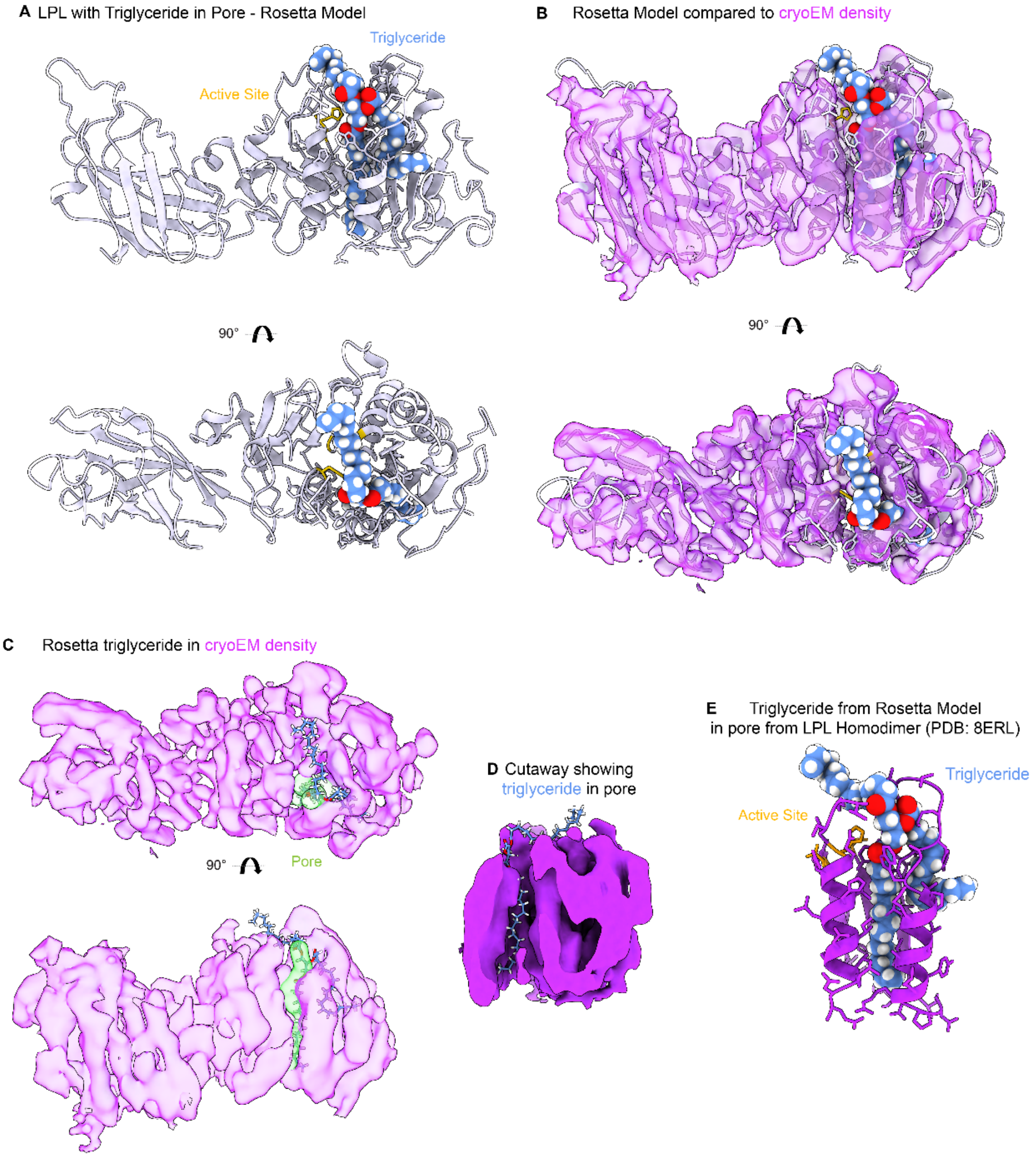

Supplementary Figure 5 – Modeling of a triglyceride substrate into an LPL homodimer subunit

A) The results of PyRosetta modeling for LPL (white) with a triglyceride (light blue) ligand. The sn-1 acyl chain is in the hydrophobic pore, the sn-2 acyl chain is in a pocket to the side of the active site, the sn-3 acyl chain is in the hydrophobic pocket revealed by opening of the lid peptide. The starting and final models for the triglyceride fit are available as Supplementary Data 7-8. B) Comparison of the PyRosetta model to the cryoEM density (transparent purple) C) The triglyceride from the PyRosetta model overlaid with the cryoEM map and identified pore (transparent lime green). D) A slice of the LPL density containing the triglyceride model showing the position of the sn1 chain in the pore and the sn-3 acyl chain in the upper hydrophobic pocket. E) The triglyceride fit in the pore residues from the LPL homodimer.

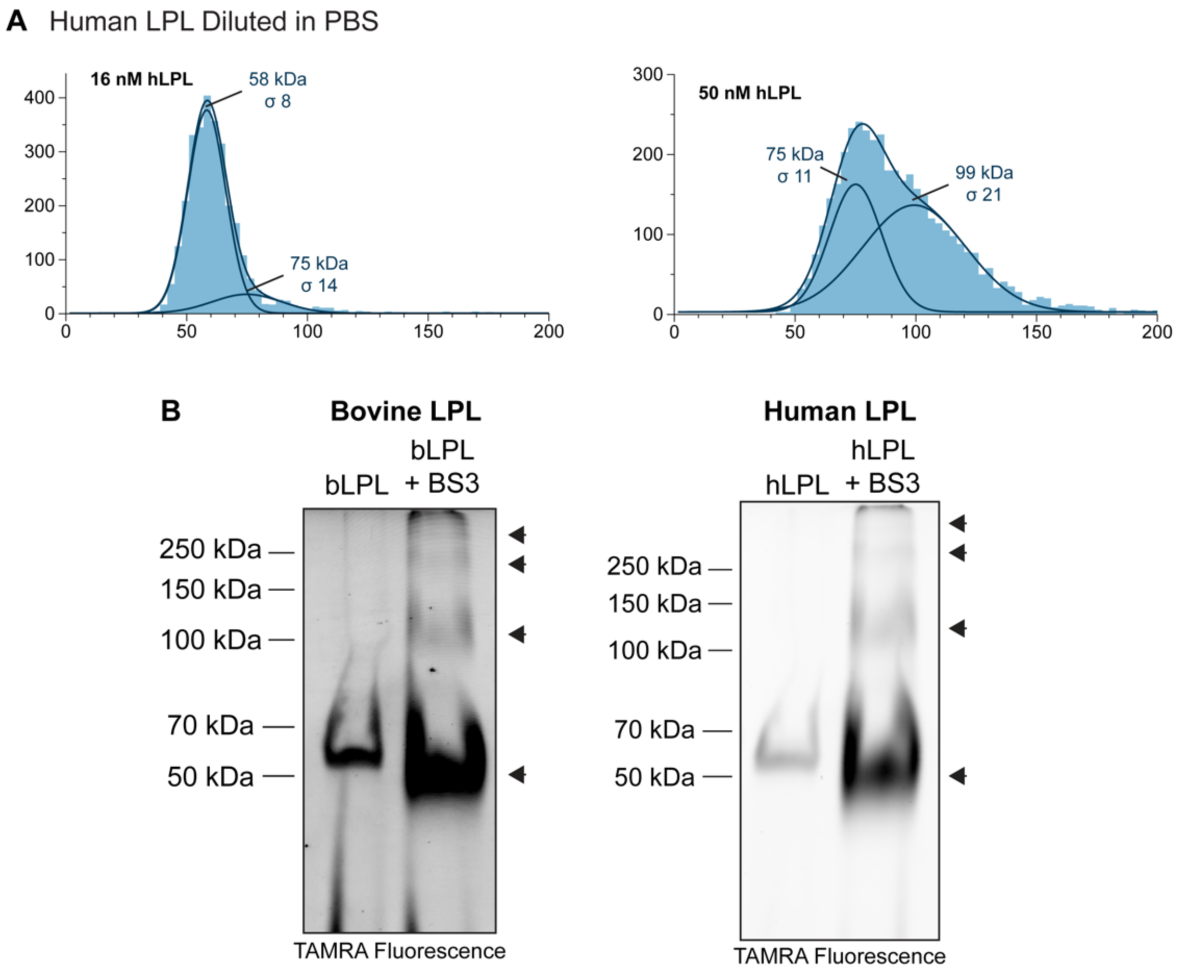

Supplementary Figure 6 – Human LPL forms the same oligomers as bovine LPL

A) Human LPL (hLPL) (furin resistant mutant) was analyzed with mass photometry at 2 concentrations, 16 nM and 50 nM. Histogram of mass distributions were fit with multiple gaussians to determine an average molecular weight (kDa) and the width of the standard deviation of the fitted gaussian is given by sigma (σ). LPL monomer theoretical molecular weight = 50.5 kDa. At 16 nM hLPL the primary peak corresponds to the molecular weight of an LPL monomer (58 kDa). At 50 nM the predominate peak has shifted to 99 kDa, corresponding to a dimer of LPL. A lower molecular weight shoulder is seen (75 kDa), suggesting the shift to a 100% dimerized population is incomplete. B) Crosslinking of both bovine LPL (bLPL) and hLPL with bis(sulfosuccinimidyl)suberate (BS3) analyzed by TAMRA-FP serine-hydrolyase probe fluorescence shows the appearance of at least 4 oligomeric species (arrows). Control uncrosslinked protein is in the first lane for comparison. BS3 crosslinking experiments were performed in triplicate. Source data are provided as a Source Data file.

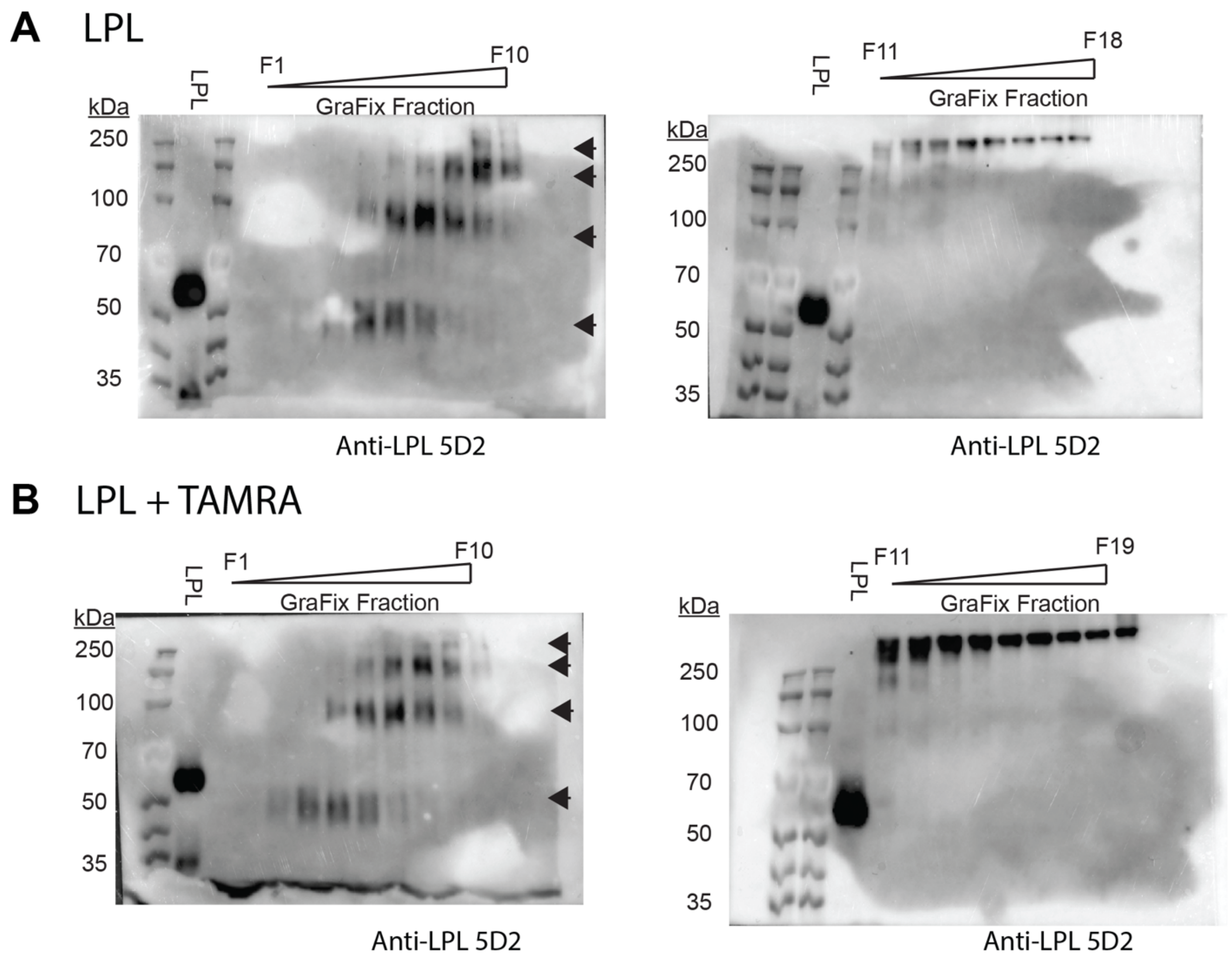

Supplementary Figure 7 – TAMRA labeling does not alter LPL oligomeric distribution

Addition of TAMRA-FP serine hydrolase probe (TAMRA) does not alter LPL oligomer distribution in GraFix. A) LPL crosslinked by GraFix has at least 4 distinct LPL oligomers (black arrows). B) LPL incubated with TAMRA and then run on GraFix shows the same oligomer formation. Western blots were developed with the LPL 5D2 antibody. Small differences in the oligomer distribution can be attributed to fractionating by hand. We subsequently used TAMRA fluorescence to identify LPL bands for downstream mass photometry due to its increased sensitivity and speed. GraFix crosslinking experiments were performed in triplicate.

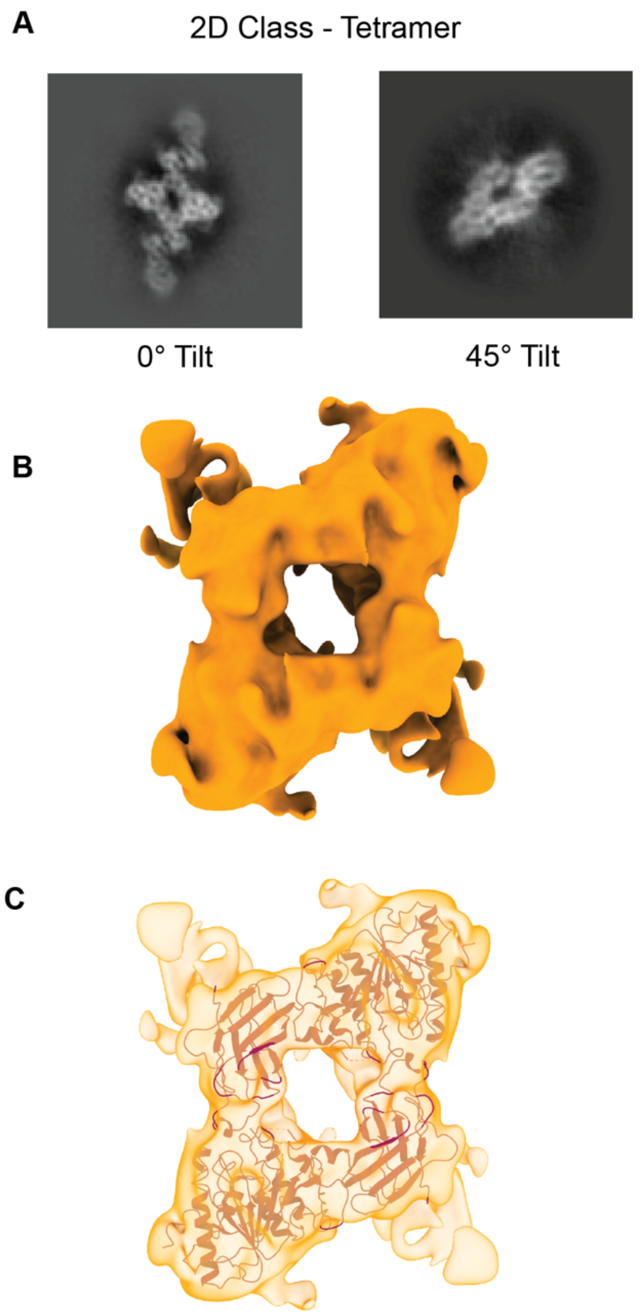

Supplementary Figure 8 – A subset of particles formed an apparent LPL tetramer

A) Some protein particles in our cryoEM data were observed adopting an apparently tetrameric oligomer of LPL – also in a preferred orientation – the center 2 interacting LPL subunits were the least mobile portion of the complex and therefore the easiest to resolve in 2D classification. We also observed the center of the tetramer in our tilted data. B) We generated a 3D model using cryosparc with the tetramer particles from selected 2D classes. C) The LPL inactive dimer subunit of the LPL helix structure (PDB 6U7M) fits very well into the density suggesting that this tetramer interaction may be mediated by the 2 center LPL subunits adopting a structure like the inactive LPL dimer found in the helix.

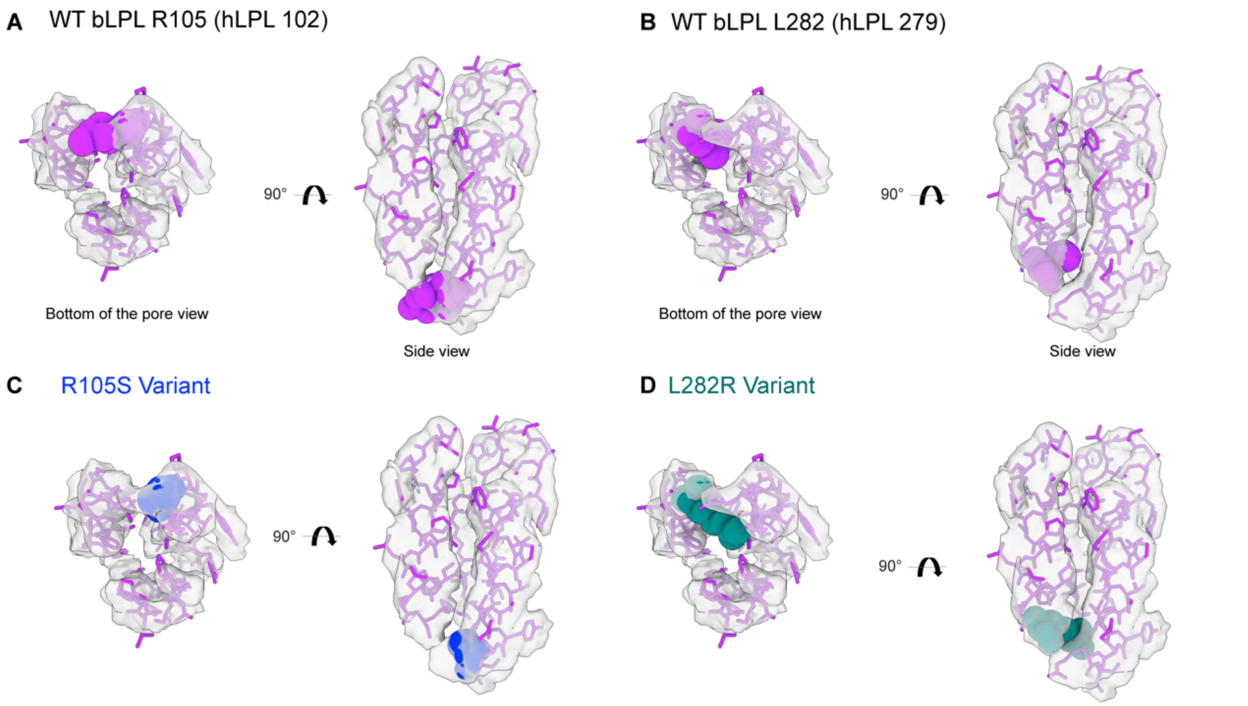

Supplementary Figure 9 – Modeling of LPL variants that have been shown to cause a defect in long acyl chain substrate hydrolysis

A) The residues (purple) and density (transparent gray) corresponding to the pore of LPL with R105 residue shown as a sphere representation (this corresponds to R102 in human LPL). B) The L282 residue shown as a sphere representation (this corresponds to L279 in human LPL). C) Modeling of the R105S variant (blue). D) Modeling of the L282R variant (teal).
