# Supplementary material for: Structure of dimeric lipoprotein lipase reveals a pore adjacent to the active site
Source: Nat Commun. 2023 May 4;14:2569. doi: 10.1038/s41467-023-38243-9 (PMC10160067; doi:10.1038/s41467-023-38243-9)
Supplement: Supplementary file 3 — Description of Additional Supplementary Files [file 41467_2023_38243_MOESM3_ESM.pdf]

Supplementary Movie 1

A movie of a single LPL subunit morphing back and forth from the structure of LPL in the LPL/GPIHBP1 crystal structure (PDB 6OB0) to an LPL subunit from the LPL homodimer (PDB 8ERL). The opening of the active site pore in the LPL homodimer is the final state of the movie. This morph is not meant to represent the physiological movement that might occur during opening of the LPL pore, but meant to demonstrate the difference between these two structures of LPL solved by different methods, one with an active site inhibitor (6OB0) and one with an open pore (8ERL).

Supplementary Movie 2

A movie of a single LPL subunit morphing back and forth from the structure of LPL from the LPL helical oligomer (PDB 6U7M) to an LPL subunit from the LPL homodimer (PDB 8ERL). The opening of the active site pore in the LPL homodimer is the final state of the movie. This morph is meant to demonstrate that the movement observed in supplementary movie 1 is similar to what we see when comparing two structures – one inactive (6U7M) and one active (8ERL) - that were both solved by cryoEM.

Supplementary Movie 3

A movie of a single LPL subunit morphing back and forth from the structure of LPL forth from the structure of LPL in the LPL/GPIHBP1 crystal structure (PDB 6OB0) to from the LPL helical oligomer (PDB 6U7M). This morph is meant to demonstrate that the movement observed in supplementary movies 1 is not necessarily a result of the technique or resolution, but the shifts observed in the structure going from an inactive to active state. This morph compares an inhibited structure (6OB0) to an inactive oligomer (6U7M)

Supplementary Data 1

This PDB file is the starting file for the PyRosetta fitting of palmitate (PLM), a saturated acyl chain with 16 carbons (C16:0), into the hydrophobic pore seen in the LPL homodimer (PDB 8ERL). The acyl chain was docked into the empty pore and relaxed using Coot.

Supplementary Data 2

This PDB file is the result of PyRosetta fitting of PLM (C16:0) into the hydrophobic pore seen in the LPL homodimer (PDB 8ERL).

Supplmentary Data 3

This PDB file is the starting file for the PyRosetta fitting of oleate (OLA), a monounsaturated acyl chain with 18 carbons (C18:1), into the hydrophobic pore seen in the LPL homodimer (PDB 8ERL). The acyl chain was docked into the empty pore and relaxed using Coot.

Supplementary Data 4

This PDB file is the result of PyRosetta fitting of OLA (C18:1) into the hydrophobic pore seen in the LPL homodimer (PDB 8ERL).

Supplementary Data 5

This PDB file is the starting file for the PyRosetta fitting of linoleate (EIC), a doubly unsaturated acyl chain with 18 carbons (C18:2), into the hydrophobic pore seen in the LPL homodimer (PDB 8ERL). The acyl chain was docked into the empty pore and relaxed using Coot.

Supplementary Data 6

This PDB file is the result of PyRosetta fitting of EIC (C18:2) into the hydrophobic pore seen in the LPL homodimer (PDB 8ERL).

Supplementary Data 7

This PDB file is the starting file for the PyRosetta fitting of a triglyceride (TGL), which is composed of a glycerol backbone with three saturated 18 carbon chains (C18:0) into the LPL structure (PDB 8ERL). The acyl chain in the sn-1 position was fit into the hydrophobic pore. The ester bond of the acyl chain to the glycerol backbone, where hydrolysis occurs, was oriented toward the active site residues. The sn-1 acyl chain was relaxed into the pore using Coot. The sn-2 and sn-3 acyl chains were relaxed into the structure based on their orientation following alignment with the active site. The sn-2 chain sits in a pocket located opposite the active site, while the sn-3 chain sits in the hydrophobic pocket revealed by the lid peptide opening.

Supplementary Data 8

This PDB file is the result of PyRosetta fitting of TGL into the hydrophobic pore seen in the LPL homodimer (PDB 8ERL).

Supplementary Data 9

PDBePISA analysis results showing residues involved in interaction interfaces for the LPL helix (C-terminal to C-terminal and C-terminal to N-terminal), LPL homodimer (C-terminal to C-terminal), LPL/GPIHBP1 heterodimer (C-terminal to GPIHBP1). Highlighted residues indicate the overlap with the interface listed at the bottom of the table. Residue numbering for both human and bovine LPL is included to facilitate comparison.
