# Supplementary material for: Structure of dimeric lipoprotein lipase reveals a pore adjacent to the active site
Source: Nat Commun. 2023 May 4;14:2569. doi: 10.1038/s41467-023-38243-9 (PMC10160067; doi:10.1038/s41467-023-38243-9)
Supplement: Supplementary file 16 — Reporting Summary [file 41467_2023_38243_MOESM16_ESM.pdf]

nature portfolio

Corresponding author(s): Saskia Neher
Last updated by author(s): 4/18/2023

Reporting Summary

Nature Portfolio wishes to improve the reproducibility of the work that we publish. This form provides structure for consistency and transparency in reporting. For further information on Nature Portfolio policies, see our Editorial Policies and the Editorial Policy Checklist.

Statistics

For all statistical analyses, confirm that the following items are present in the figure legend, table legend, main text, or Methods section.

n/a	Confirmed	
☐	☒	The exact sample size (*n*) for each experimental group/condition, given as a discrete number and unit of measurement
☐	☒	A statement on whether measurements were taken from distinct samples or whether the same sample was measured repeatedly
☐	☒	The statistical test(s) used AND whether they are one- or two-sided<br>*Only common tests should be described solely by name; describe more complex techniques in the Methods section.*
☒	☐	A description of all covariates tested
☒	☐	A description of any assumptions or corrections, such as tests of normality and adjustment for multiple comparisons
☐	☒	A full description of the statistical parameters including central tendency (e.g. means) or other basic estimates (e.g. regression coefficient) AND variation (e.g. standard deviation) or associated estimates of uncertainty (e.g. confidence intervals)
☐	☒	For null hypothesis testing, the test statistic (e.g. F, t, r) with confidence intervals, effect sizes, degrees of freedom and P value noted<br>*Give P values as exact values whenever suitable.*
☒	☐	For Bayesian analysis, information on the choice of priors and Markov chain Monte Carlo settings
☒	☐	For hierarchical and complex designs, identification of the appropriate level for tests and full reporting of outcomes
☒	☐	Estimates of effect sizes (e.g. Cohen's *d*, Pearson's *r*), indicating how they were calculated

Our web collection on statistics for biologists contains articles on many of the points above.

Software and code

Policy information about availability of computer code

Data collection	SerialEM 3.8, Refeyen AcquireMP 2.2 and 2.3, SoftMax Pro 5
Data analysis	cryoSPARC v3, Topaz 0.2.3, UCSF ChimeraX 1.5, Refeyen Discover MP 2.2, Origin 2022b (9.95), DataGraph 5.0, PHENIX 1.20.1, Coot 0.9.5, PyRosetta 4 2023.05 and Online servers: 3DFSC 3.0, PDBePISA 1.52, MOLEonline 2.5

For manuscripts utilizing custom algorithms or software that are central to the research but not yet described in published literature, software must be made available to editors and reviewers. We strongly encourage code deposition in a community repository (e.g. GitHub). See the Nature Portfolio guidelines for submitting code & software for further information.

Data

Policy information about availability of data

All manuscripts must include a data availability statement. This statement should provide the following information, where applicable:

- Accession codes, unique identifiers, or web links for publicly available datasets
- A description of any restrictions on data availability
- For clinical datasets or third party data, please ensure that the statement adheres to our policy

The data that support this study are available from public repositories or in the source data file. The Cryo-EM density map was deposited in the Electron Microscopy Data Bank (EMDB) under accession number 28554 [https://www.ebi.ac.uk/pdbe/entry/emdb/EMD-28554]. Model coordinates have been deposited in the Protein Data Bank (PDB) under accession number 8ERL [https://doi.org/10.2210/pdb8ERL/pdb] (LPL homodimer). Other structures used in this study were obtained from

the PDB with accession codes 6U7M [https://doi.org/10.2210/pdb6U7M/pdb] (LPL helix), PDB 6OB0 [https://doi.org/10.2210/pdb6OB0/pdb] (LPL with GPIHBP1), 1LPA [https://doi.org/10.2210/pdb1LPA/pdb] (PTL/procolipase complex), and 1ETH [https://doi.org/10.2210/pdb1ETH/pdb] (porcine PTL). Map 20673 [https://www.ebi.ac.uk/emdb/EMD-20673] (LPL helix) from the EMDB was also used. Models for LPL/ligand complexes are available as Supplementary files 1 through 8, including before and after minimization with PyRosetta. Interface analysis results are available as Supplementary Data 9. Source data are provided with this paper for each figure where relevant raw data exists.

Human research participants

Policy information about studies involving human research participants and Sex and Gender in Research.

Reporting on sex and gender	N/A
Population characteristics	N/A
Recruitment	N/A
Ethics oversight	N/A

Note that full information on the approval of the study protocol must also be provided in the manuscript.

Field-specific reporting

Please select the one below that is the best fit for your research. If you are not sure, read the appropriate sections before making your selection.

☒ Life sciences ☐ Behavioural & social sciences ☐ Ecological, evolutionary & environmental sciences

For a reference copy of the document with all sections, see nature.com/documents/nr-reporting-summary-flat.pdf

Life sciences study design

All studies must disclose on these points even when the disclosure is negative.

Sample size	From initial data collections of ~3000 micrographs we estimated that we would need three times this amount with approximately two-thirds of the movies collected at a tilt angle. Therefore we set out to collect ~4000 movies per tilt with the final number being - 13,748 micrographs. The final structure was solved from 527,205 particles, which were determined through 2D and 3D classification.
Data exclusions	Micrographs with low resolution CTF fits and/or high total full-frame motion distance were excluded. 2D classes were excluded that did not show clear density for the entire LPL dimer. 2D classes representing LPL tetramers were also excluded. This resulted in 1,035,101 particles. Ab initio models and 3D classes with the most stretching in their maps - resulting from anisotropy - were excluded. This resulted in the 527,205 particles used to solve the structure.
Replication	Mass photometry, Gradient fixation, crosslinking, protein purification, grid freezing, 2D classification, and activity assays were replicated a minimum of 3 times. All replication attempts yielded the same result, except for grid freezing, which often resulted in ice heterogeneity affecting ability to image the grids. PyRosetta theoretical modeling was performed twice per ligand with the same results.
Randomization	The particles used in the final structure were randomly split into 2 batches that were independently refined during 3D refinement in cryoSPARC. We did not split any other samples into experimental groups for data analysis purposes.
Blinding	The particles were split by cryoSPARC into the two random groups and the researchers did not know which particles were in which group. No other blinding was needed for these experiments because we were not sorting data into groups or making qualitative assessments.

Reporting for specific materials, systems and methods

We require information from authors about some types of materials, experimental systems and methods used in many studies. Here, indicate whether each material, system or method listed is relevant to your study. If you are not sure if a list item applies to your research, read the appropriate section before selecting a response.

Materials & experimental systems

n/a	Involved in the study	
☐	☒	Antibodies
☒	☐	Eukaryotic cell lines
☒	☐	Palaeontology and archaeology
☒	☐	Animals and other organisms
☒	☐	Clinical data
☒	☐	Dual use research of concern

Methods

n/a	Involved in the study	
☒	☐	ChIP-seq
☒	☐	Flow cytometry
☒	☐	MRI-based neuroimaging

Antibodies

Antibodies used

anti-Lipoprotein Lipase clone 4-1a (Millipore, MABS1270) at 1:5000, anti-Lipoprotein lipase 5D2 (Abcam ab93898) at 1:250, Goat anti-mouse IgG-Horseradish peroxidase (HRP) (Southern Biotech, 11030-05) at 1:5000

Validation

All antibodies used in this study are commercially available.
anti-Lipoprotein Lipase clone 4-1a = manufacturer states species reactivity include bovine, human, mouse, and rat LPL. AAntibody validation statement is available on the manufacturer's website. Antibody was validated in the lab for western blotting by blotting known amounts of purified bovine LPL.
anti-Lipoprotein lipase 5D2 = manufacturer states bovine LPL was used to generate antibody and species reactivity include human, mouse, and cat LPL. Antibody validation statement is available on the manufacturer's website. Antibody was validated in the lab for western blotting by blotting known amounts of purified bovine LPL.
anti-mouse IgG-HRP = manufacturer states antibody reacts with heavy chains of mouse 1gG1, 1gG2a, IgG2b, IgG2c, and IgG3. Antibody validation statement is available on the manufacturer's website.
